# Differential modulation of host genes in the kidney of brown trout *Salmo trutta* during sporogenesis of *Tetracapsuloides bryosalmonae* (Myxozoa)

**DOI:** 10.1186/s13567-014-0101-z

**Published:** 2014-10-04

**Authors:** Gokhlesh Kumar, Ahmed Abd-Elfattah, Mansour El-Matbouli

**Affiliations:** Clinical Division of Fish Medicine, Department for Farm Animals and Veterinary Public Health, University of Veterinary Medicine, Veterinärplatz 1, 1210 Vienna, Austria

## Abstract

*Tetracapsuloides bryosalmonae* (Myxozoa) is the causative agent of proliferative kidney disease in various species of salmonids in Europe and North America. In Europe, spores of *T. bryosalmonae* develop in the kidney of infected brown trout *Salmo trutta* and are released via urine to infect the freshwater bryozoan *Fredericella sultana*. The transcriptomes of kidneys of infected and non-infected brown trout were compared by suppressive subtractive hybridization. Differential screening and a subsequent NCBI BLAST analysis of expressed sequence tags revealed 21 transcripts with functions that included cell stress and cell growth, ribonucleoprotein, signal transduction, ion transporter, immune response, hemoglobin and calcium metabolisms. Quantitative real time PCR was used to verify the presence of these selected transcripts in brown trout kidney at sporogonic stages of *T. bryosalmonae* development. Expression of cold-inducible RNA-binding protein, cyclin-dependent kinase inhibitor 2A, prothymosin alpha, transforming protein RhoA, immunoglobulin light chain and major histocompatibility complex class I were up-regulated significantly in infected brown trout. Expression of both the hemoglobin subunit beta and stanniocalcin precursor were down-regulated significantly in infected brown trout. This study suggests that cell stress and cell growth processes, signal transduction activities, erythropoiesis and calcium homeostasis of the host are modulated during sporogonic stages of parasite development, which may support the sporogenesis of *T. bryosalmonae* in the kidney of brown trout.

**Electronic supplementary material:**

The online version of this article (doi:10.1186/s13567-014-0101-z) contains supplementary material, which is available to authorized users.

## Introduction

*Tetracapsuloides bryosalmonae* belongs to the metazoan phylum Myxozoa (class: Malacosporea) and causes proliferative kidney disease (PKD) in various species of salmonids [1-3]. This parasite is found in Europe and North America and can lead to severe losses in rainbow trout *Oncorhynchus mykiss* and brown trout *Salmo trutta* farms [4] and the associated economic impacts of this disease make it an important factor for aquaculture [5]. Additionally, PKD is suspected of contributing to population declines of wild brown trout and other salmonids [6,7].

Fish species most affected by *T. bryosalmonae* belong to the genera *Salmo*, *Oncorhynchus* and *Salvelinus* [4,8]. Other susceptible hosts include grayling *Thymallus thymallus* and the non-salmonid Northern Pike *Esox lucius* in which extrasporogonic stages similar to those of *T. bryosalmonae* have been found [9,10]. Only brown trout and brook trout *Salvelinus fontinalis* can transmit the parasite back to its obligate invertebrate host, bryozoans [11,12]. Sporogonic stages of *T. bryosalmonae* were seen in the renal tubules of brown trout infected with the European strain of *T. bryosalmonae* at different time points that could transmit the parasite to bryozoan colonies [13]. Furthermore, we verified the persistence of *T. bryosalmonae* in chronically infected brown trout and their ability to infect the bryozoan up to 104 weeks post exposure (wpe) [14].

Suppression subtractive hybridization (SSH) can identify transcripts that are differentially either up- or down-regulated in two RNA samples [15]. SSH was used to identify differential expression of immune relevant genes in resistant and susceptible strains of Atlantic salmon *Salmo salar* infected with the monogenean *Gyrodactylus salaris* [16]. In myxozoan parasite research, SSH has been used to study activated and inactivated spores of *Myxobolus cerebralis* [17], and to identify differentially up- or down-regulated genes in the head kidney and intestine of susceptible and resistant gilthead sea bream *Sparus aurata* infected with *Enteromyxum leei* [18]. To date, nothing is known about differentially up- or down-regulated transcripts in response to the development of *T. bryosalmonae* in the kidney of the brown trout host. In this study, we compared the transcriptomes of kidneys of infected and non-infected brown trout by suppressive subtractive hybridization. We discovered transcripts differentially expressed in the kidneys of brown trout during sporogonic stages of parasite development. Additionally, we quantified relative expression of the target transcripts in the kidney samples of brown trout. These gene expression data demonstrate the differential modulation of host genes during sporogonic stages of *T. bryosalmonae* and help improve our understanding of renal cell mechanisms and regulations.

## Materials and methods

### Ethics statement

This study was approved by the institutional ethics committee of the University of Veterinary Medicine Vienna and the national authority, according to §26 of the Austrian Law for Animal Experiments, Tierversuchsgesetz 2012 under approval number GZ 68.205/0247-II/3b/2011.

### Experimental design and fish sampling

Prior to the experiment, certified specific pathogen-free (SPF) brown trout stock was sampled randomly and tested by quantitative real time PCR (qPCR) to confirm the absence of *T. bryosalmonae* according to Grabner and El-Matbouli [19]. Prior to infection, SPF 60 brown trout (mean length 5.5 ± 0.5 cm, mean weight 2.3 ± 0.5 gm) were transferred to a small aquarium filled with 25 liters volume of water and the water supply was stopped for 24 h. Free *T. bryosalmonae* spores in suspension, released from 12 mature sacs of parasite from laboratory infected *Fredericella sultana* colonies, were added to the aquarium, which was then maintained with vigorous aeration for 24 h at 16.5 ± 1 °C. After infection, fish were distributed between 3 aquaria, 20 fish per aquarium filled with 100 liters volume of water. Additional 30 brown trout were held as a non-infected control in separate aquaria. Fish were maintained at 16.5 ± 1 °C with 3 liters per minute running water flow rate and fed everyday with 1% of the body weight. No mortalities of fish occurred during initial exposure, and only 3 fish died between 11 and 12 wpe. Posterior kidneys were sampled from both infected (*n* = 10 fish) and control groups (*n* = 5 fish) at 6, 8, 10 and 12 wpe and the rest of the fish at 14 and 17 wpe. Tissue from each fish was divided into 2 portions, 1 fixed in 10% neutral buffered formalin for histology, and 1 in RNA*later* (Sigma, Steinheim, Germany) for gene expression study.

### mRNA preparation

**Figure 1:**
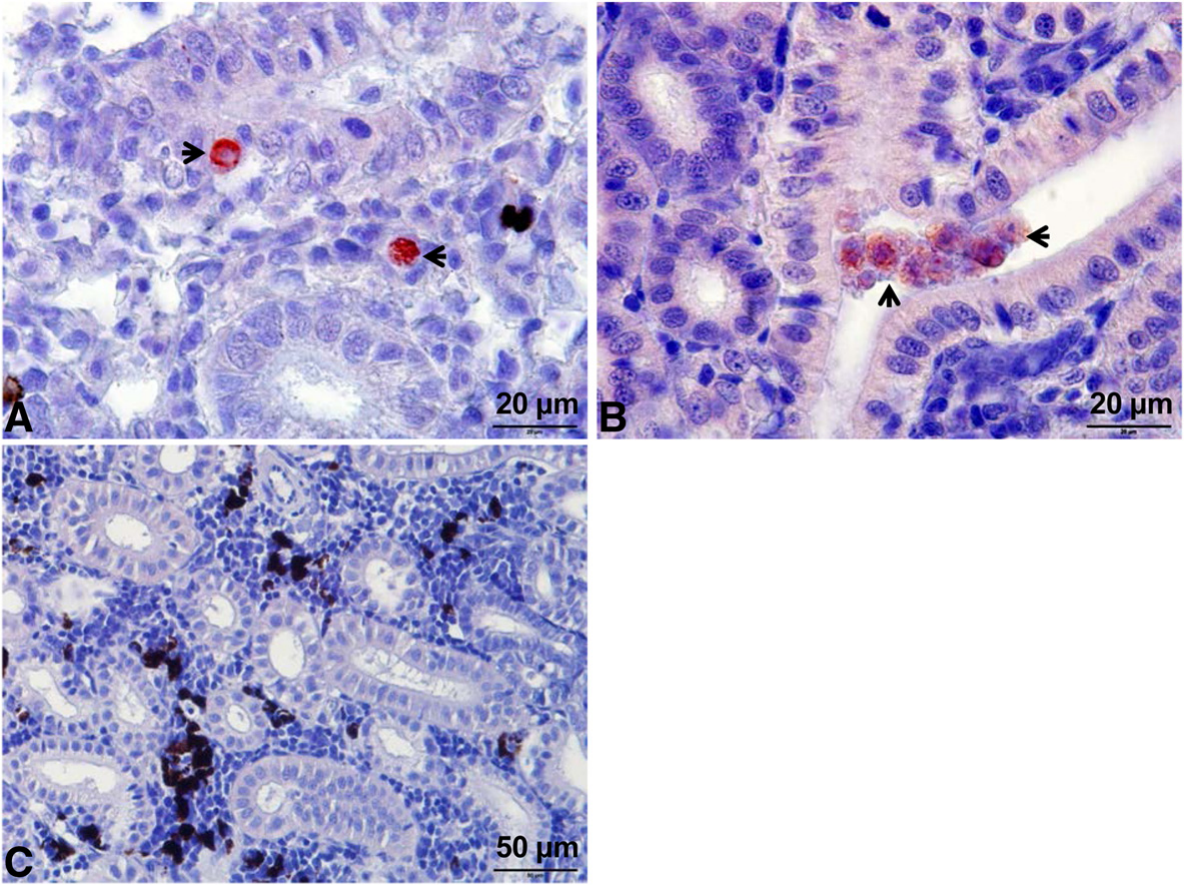
***Tetracapsuloides bryosalmonae*** **stages in the kidney of brown trout. (A)** Interstitial pre-sporogonic stages (arrows); **(B)** Renal tubule filled with numerous sporogonic parasite stages (arrows); **(C)** Non-infected brown trout kidney control. Parasite stages were visualized by immunohistochemistry using monoclonal antibody against *T. bryosalmonae* and counterstained with haematoxylin.

The optimal time point (8–10 wpe) for the SSH assay was determined by the presence of numerous intra-luminal stages of *T. bryosalmonae* with low numbers of interstitial pre-sporogonic stages in the kidney of brown trout, observed using immuno-histological examination [13]. Additionally, at 6 wpe, low numbers of pre-sporogonic stages of *T. bryosalmonae* were seen in the kidneys (Figure 1A), whereas the sporogonic stage was almost nil. Total RNA was extracted from the kidneys of 8 infected fish with high numbers of intra-luminal sporogonic stages of *T. bryosalmonae* (Figure 1B) and non-infected control fish (Figure 1C), using an RNeasy mini kit (Qiagen, Hilden, Germany). An on-column DNase (Qiagen) digestion step was included. Equal amounts of RNA (25 μg) of individual fish were pooled to even out differences in expression between individual fish. Messenger RNA were purified from the pooled RNA (200 μg) sample of all 8 fish using an Oligotex mRNA kit (Qiagen).

### Suppression subtractive hybridization

Two micrograms of mRNA were reverse transcribed into first-strand cDNA using SMARTScribe reverse transcriptase, then into second-strand cDNA by a second-strand enzyme cocktail (Clontech, Saint-Germain-en-Laye, France). PCR was used to assess the relative amount of cDNA products after double strand (ds) cDNA synthesis, using trout beta-actin gene primers [20].

The cDNA pools of infected and non-infected control brown trout were hybridized in 2 steps. A PCR-Select cDNA subtraction kit (Clontech) was used for SSH. Forward, reverse and control hybridizations were performed to create subtracted cDNA per manufacturer guidelines. The forward subtraction, infected brown trout cDNA was used as the Tester and non-infected control brown trout cDNA as the Driver. For the reverse subtraction, non-infected control brown trout cDNA was used as the Tester and infected brown trout cDNA as the Driver. The ds cDNA was digested with RsaI restriction enzyme and purified. Ligation, hybridization, and primary and secondary PCR were performed according to the manufacturer’s protocols. Finally, subtraction efficiency was evaluated to confirm the reduced abundance of SSH libraries using PCR with trout beta-actin gene primers. Subtracted secondary PCR products were cloned into a pCR4 TOPO TA cloning vector (Invitrogen, Carlsbad, USA) and transformed into *Escherichia coli* TOP10 cells to create the forward and reverse subtracted cDNA libraries. All clones were picked and tested by colony PCR using adaptor-specific nested PCR primer 1 and nested PCR primer 2R in a Clontech kit. PCR was performed as follows: 30 cycles of 94 °C for 45 s, 66 °C for 45 s and 72 °C for 90 s.

### Differential expression screening of clones

To validate and explore patterns of gene expression, differential expression screening was performed with a dot blot of PCR products from both populations, which represented differentially expressed transcripts.

For the dot blot, the DIG-High Prime DNA Labeling and Detection Starter Kit I (Roche Applied Science, Mannheim, Germany) was used. The cDNA population of subtracted forward, subtracted reverse, unsubtracted tester control and unsubtracted driver control were labeled with DIG-digoxigenin, and used as probes for the dot blot hybridization. Analysis of the DIG labeling efficiency was performed as per the manufacturer’s instructions. To denature the DNA, a final concentration of 0.4 M NaOH/10 mM EDTA was added to each of the SSH library clones amplified by the adaptor-specific PCR primers (1 and 2R), incubated at 99 °C for 10 min, then held on ice. Two microliters of each denatured PCR product was dotted onto a positively charged nylon membrane and UV cross-linked. Membranes were prehybridized in DIG Easy Hyb and then hybridized overnight at 42 °C with each of the constructed DIG-labelled cDNA probes. Hybridization and subsequent detection was performed using anti-DIG-alkaline-phosphatase conjugate and BCIP/NBT substrate following the manufacturer’s protocol. Differential expression of enriched subtracted forward and reverse cDNA libraries were analyzed and compared with unsubtracted cDNA library controls. Positive dots of subtracted forward and reverse cDNA libraries were identified as those that had different signal intensities on the probed membrane. Dots with similar intensities and weak signals were considered to be non-differentially expressed transcripts.

### Sequencing analysis

A plasmid from each positive clone of subtracting forward and reverse cDNA libraries was purified, then sequenced at LGC Genomics GmbH, Berlin, Germany. Vector and adaptor-sequences were removed from the expressed sequence tags (EST). The basic local alignment search tool (BLAST) searches were performed to identify the expressed sequence tags (EST) in the NCBI non-redundant sequence database using BLASTn and BLASTx [21]. Biological and molecular functions of transcripts were putatively identified through searches against the Gene Ontology [22], and UniProt databases [23].

### Quantitative real-time PCR

Infection in individual kidneys collected at different time points were first confirmed by immuno-histology and qPCR of *T. bryosalmonae*. Parasite load was low in kidneys at 6 wpe and high at 8–12 wpe; the details were published in our previous study [13]. Total RNA was extracted from the kidney samples using an RNeasy Mini Kit (Qiagen) according to the manufacturer’s instructions, and included an on-column DNase digestion step. cDNA was synthesized using an iScript cDNA Synthesis Kit (BIO-RAD, Hercules, USA) with 1000 ng total RNA per the user’s manual. Nine important transcripts were selected based on their functions such as cell and growth stress, signal transduction activity, protein synthesis, immune gene, heme binding protein and calcium homeostasis to verify the transcriptional level in infected brown trout at pre-sporogonic (6 wpe) and sporogonic stages (8, 10 and 12 wpe) of parasite development and non-infected control brown trout.

PCR primers specific for the 9 selected genes were designed (Table 1) using the primer design tool of NCBI Primer-BLAST software [24]. PCR assays were optimized using gradient PCR to determine the optimal annealing temperature and primer concentration. A CFX96 Touch Real-Time PCR detection system (BIO-RAD) was used to quantify gene expression levels using iQ SYBR Green Supermix (BIO-RAD). qPCR had a final volume of 20 μL, which contained 4 μL of 1:10 fold diluted cDNA, 0.4 μM of each primer, 1X SYBR Green Supermix and sterile distilled water. After 5 min of cDNA denaturation at 95 °C, 38 cycles were performed at 95 °C for 30 s, 53–62 °C for 30 s and 72 °C for 30 s. A melting-point curve was then measured, starting from 53–62 °C and increasing by 0.5 °C every 10 s up to 95 °C, to detect any non-specific PCR products. Each qPCR was performed in triplicate. Trout beta-actin [20] and elongation factor alpha 1 (EF-1α) were used as reference genes for normalization. Standard curves were constructed for target transcripts and reference genes.

**Table 1:** **Nucleotide sequence of quantitative real-time PCR primers used in this study**

**Primer name**	**Sequence (5′-3′)**	**Annealing temperature (°C)**	**Amplicon size (bp)**	**GeneBank accession no.**
CIRBP F	CATCCCTTGGCTGGCTGTAT	62	169	JZ713052
CIRBP R	GGAAATGAATGGCCGACACAC			
CDKN2AIP F	ATGGGCCAACAACGTGTTTC	56	103	JZ713054
CDKN2AIP R	GAAAACAGGGGCATCCTCCA			
PTMA-α F	GCCCCTGTAACCTCTCTCCT	55	108	BT057594
PTMA-α R	TGTGTACACGGACATTGGGT			
TP RhoA F	GTTGGTGATGGTGCTTGTGG	57	185	JZ713063
TP RhoA R	AGAGGCCGTAGTCTGTCGTA			
RPL6 F	ATGGCAGAGGGAGACAAGAA	56	146	NM_001141697
RPL6 R	GGTCTCAGTGGTCTTGGTCT			
IgL F	AGTGGAGTCCAGGCTGAAGA	57	119	AF273017
IgL R	AGGGTGGGGACACTGTTACT			
MHC-I F	CAGGTGTGCACGTTTTCCAG	57	163	JZ713068
MHC-I R	TTGGTGATGACTGCCTGTGG			
Hemoglobin F	CAGTGCTGAATAGGCGTTCTT	62	145	JZ713069
Hemoglobin R	ACACTTCAGCACCTTCGGC			
Stanniocalcin F	GCCATGACATCCCCGTTTTG	57	144	JZ713071
Stanniocalcin R	GATGTCAAACCCCACCCACT			
Beta-actin F*	ATGGAAGGTGAAATCGCC	53	260	AF157514
Beta-actin R*	TGCCAGATCTTCTCCATG			
EF-1α F	AGACAGCAAAAACGACCCCC	57	167	HF563594
EF-1α R	AACGACGGTCGATCTTCTCC			

*From Rucker and El-Matbouli [20].

### Statistical analysis

Relative expression levels of target transcripts were analyzed at each time point using a linear mixed effect model. Adjustment for multiple comparisons was performed using SIDAK’s procedure [25]. The differences between groups (infected and controls) at each single time point were analyzed using *t*-tests for independent samples with Bonferroni α-correction. Correlations between relative expression levels of target transcripts were analyzed by calculating the Pearson product–moment correlation coefficient. For all statistical tests, a *p*-value < 0.05 was regarded as significant. All statistical analyses were conducted with SPSS version-20 software.

## Results

### Identification of EST from the SSH library

Four hundred twenty-nine clones (170 forward and 259 reverse subtracted cDNA libraries) were screened using adaptor-specific PCR. PCR products indicated variation in the size of inserts in the cDNA libraries (see Additional file 1). Dot blot screening identified 86 clones (27 forward, 59 reverse) that exhibited different signal expression activity (see Additional file 2). NCBI BLAST searches show that 21/86 SSH clones were similar to genes with functions that include cellular stress, cell growth, protein biosynthesis, signal transduction, ion transporter, humoral immune response, antigen processing and presentation, hemoglobin, calcium/phosphate metabolisms and cytoskeleton organization (Tables 2 and 3), and judged as candidate transcripts. BLAST searches of the EST of these transcripts matched known nucleotide sequences from fish, with sequence similarities of 88–100%. No EST were significantly similar to hypothetical proteins. EST deposited to the GenBank dbEST database can be accessed under accession numbers: JZ713052 to JZ713072.

**Table 2:** **cDNA sequences of the transcripts revealed from the forward subtracted library**

**Transcript**	**GeneBank accession no.**	**Homolog Species**	**Nucleotide identity (%)**	**Function**
Cold-inducible RNA-binding protein	BT050401.2	*Salmo salar*	99	Cellular stress
CDKN2AIP N-terminal-like protein	NM_001141388.2	*Salmo salar*	88	Cell growth regulation
Prothymosin alpha	BT057594.1	*Salmo salar*	99	Cell proliferation
Integral membrane protein 2B	BT045039.1	*Salmo salar*	99	Membrane protein/beta-amyloid binding
Ribosomal protein L6	NM_001141697.1	*Salmo salar*	99	Ribonucleoprotein complex
26S protease regulatory subunit S10B	NM_001140882.1	*Salmo salar*	97	Proteosome complex
Elongation factor 2	BT071866.1	*Salmo salar*	97	Protein biosynthesis
Ferritin, middle subunit	BT047913.1	*Salmo salar*	98	Cellular iron ion homeostasis
NADH dehydrogenase 1 beta subcomplex subunit 6	NM_001140996.2	*Salmo salar*	98	Mitochondrial membrane respiratory chain
Cytochrome oxidase subunit 1	JX960943.1	*Salmo salar*	100	Electron transport
Rab GDP dissociation inhibitor beta	NM_001141733.1	*Salmo salar*	99	Protein transport/signal transduction
Transforming protein RhoA	BT045789.1	*Salmo salar*	99	GTPase mediated signal transduction
Gamma-secretase subunit PEN-2	BT049515.1	*Salmo salar*	99	Gamma-secretase complex/notch signaling pathway/amyloid precursor protein
Immunoglobulin light chain precursor	AF273017.1	*Salmo salar*	90	Humoral immune response
Ig kappa chain V region	BT074227.1	*Oncorhynchus mykiss*	94	Humoral immune response
IgM heavy chain 7.3	Y12456.1	*Salmo salar*	95	Humoral immune response
MHC class I	AF504016.1	*Salmo salar*	96	Antigen processing and presentation/immune response

**Table 3:** **cDNA sequences of the transcripts revealed from the reverse subtracted library**

**Transcript**	**GeneBank Accession no.**	**Homolog species**	**Nucleotide identity (%)**	**Function**
Hemoglobin subunit beta	BT058629.1	*Salmo salar*	99	Heme binding/oxygen transport
Stanniocalcin precursor	BT071934.1	*Salmo salar*	97	Calcium homeostasis/hormone activity
Carbonic anhydrase 1	NM_001124220.1	*Oncorhynchus mykiss*	99	Carbon metabolic/zinc ion binding
Tubulin alpha-1A chain	NM_001141467.1	*Salmo salar*	100	Microtubule cytoskeleton

### Comparisons of relative transcript expression levels

**Figure 2:**
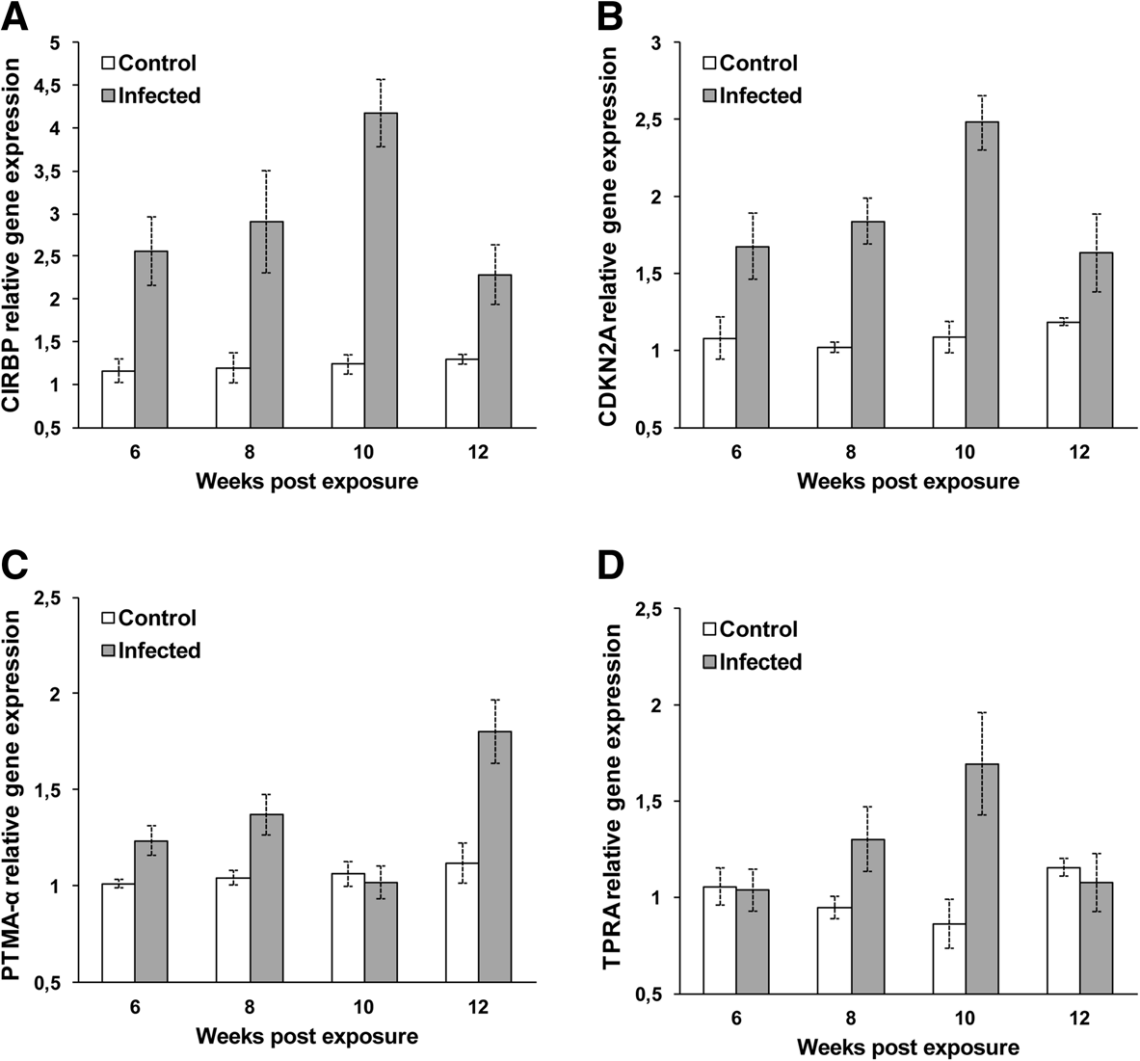
**Quantitative real-time PCR showing relative expression profiles of selected transcripts in infected and non-infected brown trout controls.** Relative gene expression changes were determined by calculating the mean expression values from the infected and control kidney samples. Each value represents the mean of three independent biological samples and error bars indicate standard deviation **(A)** Cold-inducible RNA-binding protein; **(B)** Cyclin-dependent kinase inhibitor 2A; **(C)** Prothymosin alpha; **(D)** Transforming protein RhoA.

**Figure 3:**
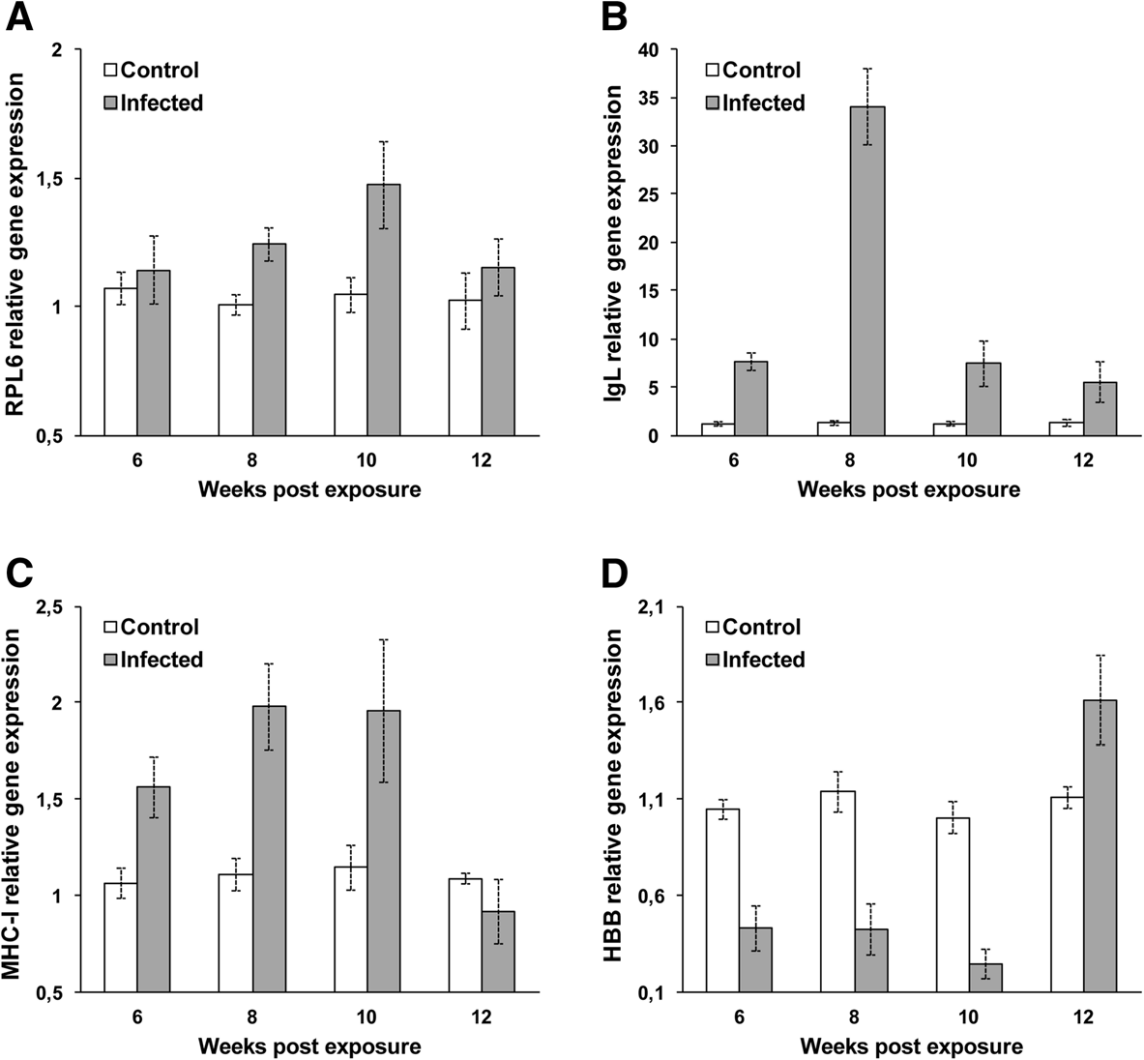
**Quantitative real-time PCR showing relative expression profiles of selected transcripts in infected and non-infected brown trout controls.** Relative gene expression details as in Figure 2. **(A)** Ribosomal protein L6; **(B)** Immunoglobulin light chain; **(C)** Major histocompatibility complex class I; **(D)** Hemoglobin subunit beta.

The mean relative gene expression of 9 tested transcripts was analyzed and compared at 6–12 wpe. Cold-inducible RNA-binding protein (CIRBP), cyclin-dependent kinase inhibitor 2A protein (CDKN2AIP), prothymosin alpha (PTMA-α), transforming protein RhoA (TPRA) and ribosomal protein L6 (RPL6) were differentially up-regulated in the kidney of brown trout during parasite development. Expression of both CIRBP and CDKN2A were up-regulated significantly (*p* < 0.003 or *p* < 0.014) in infected brown trout at all time points (Figures 2A and B). Expression of CIRBP was significantly positively correlated (r = 0.853; *p* < 0.0001) with CDKN2AIP. Expression of PTMA-α was significantly up-regulated (*p* < 0.018) in infected brown trout at 6, 8 and 12 wpe (Figure 2C). Expression of TPRA was significantly up-regulated (*p* < 0.028 or *p* < 0.003) in infected brown trout at 8 and 10 wpe but not at 12 wpe (*p* = 0.38) (Figure 2D). Expression of RPL6 was up-regulated in brown trout at 8–12 wpe but up-regulation was significant at 8 and 10 wpe (*p* < 0.037 or *p* < 0.023) (Figure 3A).

Humoral immune response and antigen presentation genes were expressed differentially in the kidney of brown trout during development of the parasite. The expression of immunoglobulin light chain (IgL) exhibited a significant positive correlation (r = 0.647; *p* < 0.008) with major histocompatibility complex class I (MHC-I). Expression of IgL was up-regulated highly significantly (*p* < 0.0001 or *p* < 0.038) in infected brown trout at all time points (Figure 3B). Expression of MHC-I was up-regulated significantly (*p* < 0.002 or *p* < 0.024) in infected brown trout at 6–10 wpe but it was not at 12 wpe (*p* = 0.223) (Figure 3C).

**Figure 4:**
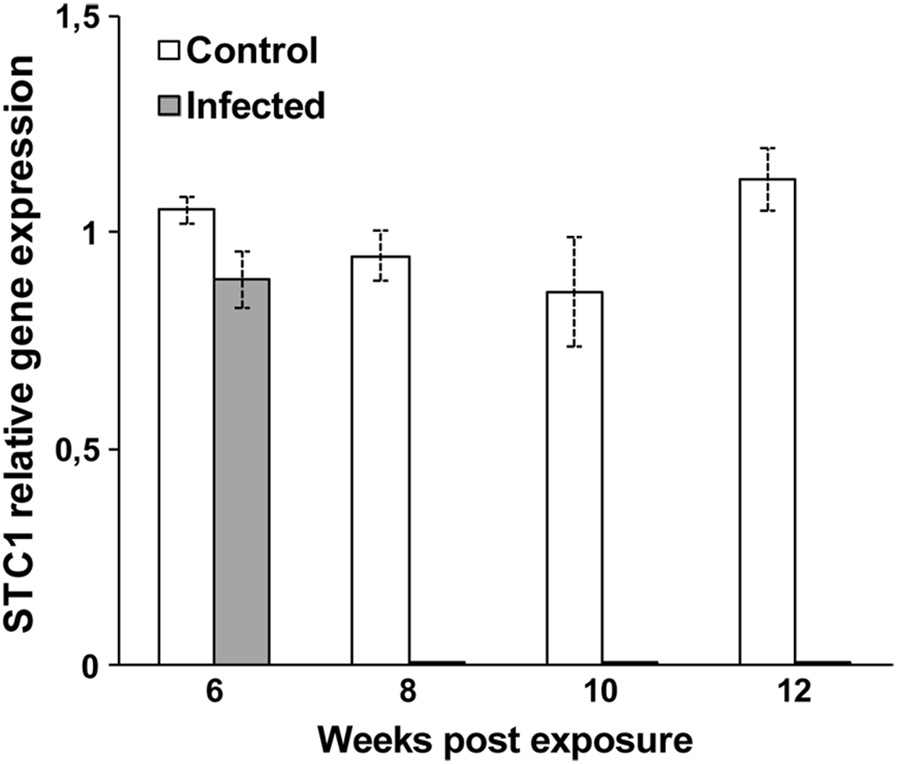
**Quantitative real-time PCR showing relative expression profiles of selected transcripts in infected and non-infected brown trout controls.** Relative gene expression details as in Figure 2. Stanniocalcin precursor.

Hemoglobin subunit beta (HBB) and stanniocalcin precursor (STC) were differentially down-regulated in the kidney of brown trout during development of the parasite. Expression of HBB was significantly down-regulated (*p* < 0.002 or *p* < 0.0001) in infected brown trout at 6–10 wpe and significantly up-regulated (*p* < 0.015) at 12 wpe (Figure 3D). Expression of STC was highly significantly down-regulated (*p* < 0.008) at 6 wpe and undetectable at 8–12 wpe (Figure 4).

## Discussion

Previous studies have examined cellular responses and immune genes in the kidney of rainbow trout naturally infected with the European strain of *T. bryosalmonae* [26-29] but nothing is known about the brown trout. The parasite spores develop in the kidney tubules of infected brown trout and are released via urine to infect bryozoan *Fredericella sultana* [11-13]. To date, nothing is known about host transcript regulation during the development of sporogonic stages of *T. bryosalmonae*. Herein we report the first gene expression study of SSH identified transcripts in the kidney of brown trout during sporogonic stages of the European strain of *T. bryosalmonae* development. In this study, we identified 21 differentially expressed transcripts, which have functions such as cellular stress, cell growth, protein biosynthesis, signal transduction, ion transporter, humoral immune response, antigen processing and presentation, hemoglobin and calcium/phosphate metabolisms. At 6 wpe (low number of pre-sporogonic stages and low parasite load), tested transcripts except hemoglobin and stanniocalcin were up-regulated. Afterwards, at 8–10 wpe (when the sporogonic stages and parasite load were increased in the kidney), the expression levels of tested transcripts (CIRBP, CDKN2A, TPRA and MHC-I) were reached to their highest levels (Figures 2 and 3). The significant transcriptional up-regulation and down-regulation of these host gene responses that we observed, suggests that they may play a role in the development of the parasite sporogonic stages in kidney tubules of the brown trout.

### Cell stress, cell growth regulation and cell proliferation

Cell stress, cell proliferation and cell growth regulation genes which code for proteins such as cold-inducible RNA-binding protein, CDKN2AIP and PTMA-α, were differentially up-regulated in the kidneys of brown trout infected with *T. bryosalmonae*. CIRBP is a stress protein that plays a critical role in protective cellular activities and is linked with a wide range of molecular changes in response to stress [30]. CIRBP is up-regulated in the skin mucus of Atlantic cod *Gadus morhua* infected with *Vibrio anguillarum* [31]. We found that expression of CIRBP was up-regulated significantly in brown trout at sporogonic stages of the parasite. Cellular stress responses may therefore produce proteins that are involved in various cellular processes such as transcription, translation, and DNA recombination during infection by the parasite.

CDKN2AIP was up-regulated significantly in the kidney of brown trout at 6–10 wpe. CDKN2A, also known as p16, is a suppressor protein that plays an important role in the control of cell cycle regulation during G1-S phases [32]. CDKN4BIP is up-regulated in the fins of Hofer strain rainbow trout infected with *Myxobolus cerebralis* [33] and is up-regulated in naïve juvenile pink salmon *Oncorhynchus gorbuscha* infected with the salmon louse *Lepeophtheirus salmonis* [34]. Our results suggest that *T. bryosalmonae* infection stimulates expression of cell growth regulation in the kidney of brown trout, which may result in inhibition of cyclin-dependent kinases and reduction of renal cell division, which may support development of the parasite in the kidney tissue. However, functional experiments are needed to verify the precise roles of CDKN2A in the development of parasite sporogonic stages in the kidney of fish.

PTMA-α is an abundant small acidic nuclear protein, which is associated with cell proliferation, protection against apoptosis and chromatin remodeling activity [35]. Expression of PTMA-α is up-regulated in brown trout but down-regulated in rainbow trout during parasitic infection (unpublished data). We found PTMA-α was differentially up-regulated in infected brown trout. Up-regulation of PTMA-α in the kidney of infected brown trout suggests that cell proliferation and cell growth in that host do not impede sporogonic stages of *T. bryosalmonae*.

### Signal transduction

We found that signal transduction gene codes for signal regulatory protein such as TPRA, was differentially up-regulated in infected brown trout. TPRA is a small GTPase protein and part of Ras superfamily, which regulates the signal transduction pathway and actin cytoskeleton that is required during cell cycle cytokinesis [36]. Rho small GTPases are up-regulated in zebrafish *Danio rerio* during development and bacterial *Mycobacterium marinum* infection [37]. We found that the expression of TPRA was significantly up-regulated in the kidney of brown trout during *T. bryosalmonae* infection, suggesting that small GTPase mediated signal transduction may support parasite development. However, the function of small GTPase mediated signal transduction in the development of the parasite sporogonic stages in fish is not clear.

### Protein synthesis

We found that the ribosomal and protein biosynthesis gene such as RPL6, was differentially up-regulated in infected brown trout. RPL6 is a ribosomal protein that makes up the ribosomal subunits, and which is involved in the cellular process of translation and protein biosynthesis [38]. Other ribosomal proteins, such as L3 and S3, are up-regulated persistently in the intestine of gilthead sea bream in response to *E. leei* infection [18]. We found that expression of RPL6 was up-regulated in the kidney of brown trout, which may be linked to its roles in activation of translation and protein biosynthesis for higher levels of renal cell growth during the parasite infection.

### Humoral immune response, antigen processing and presentation

Immunoglobulin (Ig) and MHC-I were up-regulated in infected brown trout. Immunoglobulins, also known as antibodies, are antigen binding proteins present on the B cell membrane and produced by plasma cells that are used by the immune system to identify and neutralize micro-organisms [39]. Expression of secretory forms of IgM and IgT are markedly up-regulated in the kidney of rainbow trout infected with *T. bryosalmonae* [29]. We found that expression of Ig light chain kappa variable region was strongly up-regulated in the kidneys of brown trout concurrent with the developmental stages of *T. bryosalmonae*. Ig light chain kappa is a component of the variable region of every Ig, such as IgM [39]. Similarly, we found that MHC-I was differentially up-regulated in brown trout infected with *T. bryosalmonae*. MHC-I is a cell surface molecule, expressed by nearly all nucleated cells and involved in the presentation of antigenic peptide to cytotoxic T cells [39]. The beta-2 microglobulin component of MHC-I is up-regulated in the fin of rainbow trout Hofer strain infected with *M. cerebralis* [33]. Similarly, we found up-regulation of MHC-I in the kidney of brown trout infected with *T. bryosalmonae* at 6–10 wpe*.* Our findings suggest that despite the elevated expression of immune responses, they do not influence the development of sporogonic stages of *T. bryosalmonae* in the kidney of brown trout but up-regulation of immune genes may not always generate protective responses against some pathogens, particularly when they occur in an uncoordinated manner.

### Hemoglobin and calcium homeostasis

We found that hemoglobin subunit beta and stanniocalcin were differentially down-regulated in infected brown trout. Hemoglobin is the iron-containing oxygen-transport metalloprotein in erythrocytes [40]. Expression of HBB is down-regulated in the intestine of gilthead sea bream in response to *E. leei* infection [18]. Similarly, the ectoparasite *Cryptocaryon irritans* elicits down-regulation of hemoglobin in the liver of Asian sea bass *Lates calcarifer* [41]. We found down-regulation of HBB in the kidney of infected brown trout, which suggests that erythropoiesis was suppressed in the infected fish, which could generate parasitemia and anemia in brown trout. The parasitemia has been recently confirmed by PCR in the blood samples of *T. bryosalmonae* infected brown trout at different time points [14].

Stanniocalcin (STC) is a glycoprotein hormone involved in renal and intestine calcium/phosphate homeostasis and cell metabolism [42]. STC1 is expressed at higher levels in the human colon in response to infection with the protozoan parasite *Entamoeba histolytica* [43]. STC1 is up and down-regulated in the head kidney of rainbow trout challenged with attenuated infectious hematopoietic necrosis virus [44]. We found that the expression of STC was down-regulated significantly in the kidney of infected brown trout at 6 wpe, and undetectable 8–12 wpe, suggesting that *T. bryosalmonae* infection results in progressive down-regulation of calcium/phosphate metabolism activity in host renal cells, however, further investigations are needed to determine the function of STC, as well as their role in suppression of calcium/phosphate in fish during parasite infection.

In conclusion, we identified 21 transcripts with various functions that were differentially expressed in the kidney of brown trout infected with *T. bryosalmonae*. This study suggests that cell stress and cell growth processes, signal transduction activities, erythropoiesis and calcium homeostasis of the host are induced and modulated during the development of the pre-sporogonic stages of the parasite. In addition we found their expression levels reached to either peak or off-peak during sporogonic stages of parasite development, which may support the sporogenesis of *T. bryosalmonae* in the kidney of brown trout. Evidence in support of this was shown in rainbow trout which when infected with *T. bryosalmonae* were able to completely restore the renal structure and clear the majority of the parasite [13,45]. In contrast, chronically infected brown trout were unable to either get rid of the parasite or completely restore the renal morphology [13,14]. Therefore, the role of the transcripts identified cannot be separately discussed, since they may all be associated with the parasite development. This study provides key information for the understanding of brown trout kidney responses during the intra-luminal sporogonic stage of *T. bryosalmonae* development. Further research is needed to better understand how these candidate genes and their functions are involved in the development of intra-luminal sporogonic stages of *T. bryosalmonae* in the kidney of fish.

## Additional files

Additional file 1
**Results of electrophoresis of cDNA libraries amplified by adaptor-specific, nested primers 1 and 2R.** Image of agarose gel showing different sized inserts from randomly picked clones obtained by PCR with adaptor-specific, nested primers 1 and 2R on the cDNA library. Lane 1: GelPilot 1 kb Plus ladder (Qiagen), Lanes 2–27 PCR-amplified expressed sequence tags. Additional file 2
**Dot-blot hybridization of clones amplified by the adaptor-specific nested PCR.** Denatured PCR products were dotted onto a positively charged nylon membrane, hybridized with enriched subtracted and unsubtracted DIG-labelled cDNA probes and analyzed for differential screening. Positive dots were identified as those that had different signal intensities on the membrane. Dots with similar intensities were considered to be non-differentially expressed transcripts (A) dots of hybridized with enriched subtracted forward cDNA probes, (B) dots hybridized with unsubtracted cDNA probe controls.
