# Supplementary figures and images for: Differential modulation of host genes in the kidney of brown trout Salmo trutta during sporogenesis of Tetracapsuloides bryosalmonae (Myxozoa)
Source: Vet Res. 2014 Oct 4;45(1):101. doi: 10.1186/s13567-014-0101-z (PMC4198790; doi:10.1186/s13567-014-0101-z)

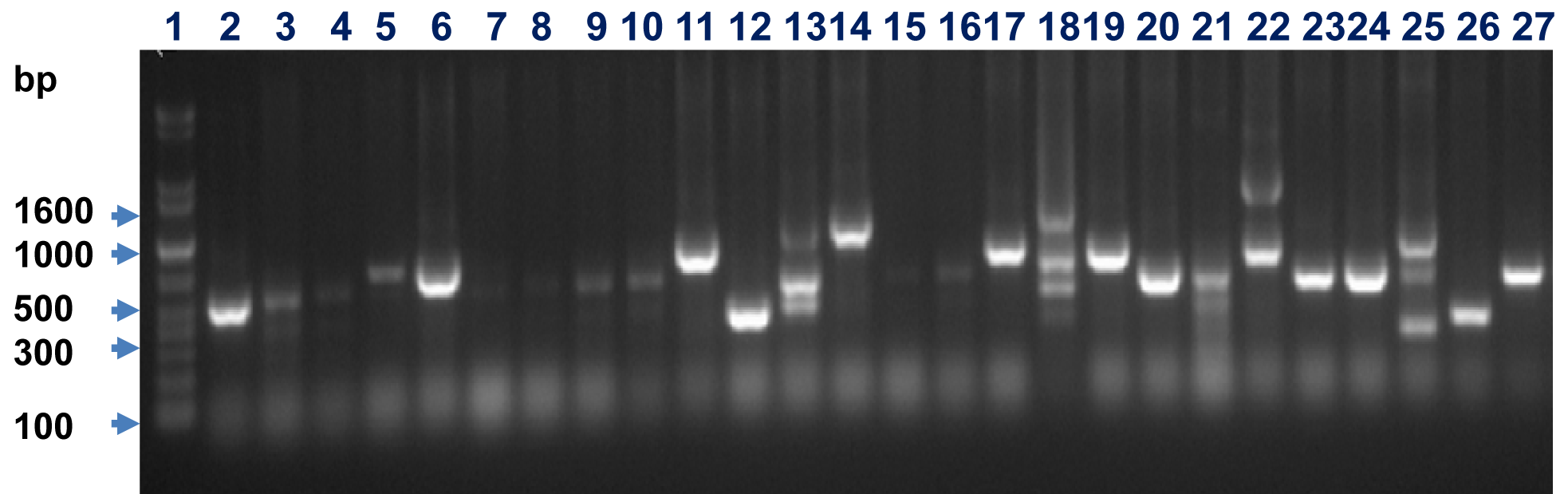

Supplement: Additional file 1 — Results of electrophoresis of cDNA libraries amplified by adaptor-specific, nested primers 1 and 2R. Image of agarose gel showing different sized inserts from randomly picked clones obtained by PCR with adaptor-specific, nested primers 1 and 2R on the cDNA library. Lane 1: GelPilot 1 kb Plus ladder (Qiagen), Lanes 2–27 PCR-amplified expressed sequence tags. [file 13567_2014_101_MOESM1_ESM.tiff]

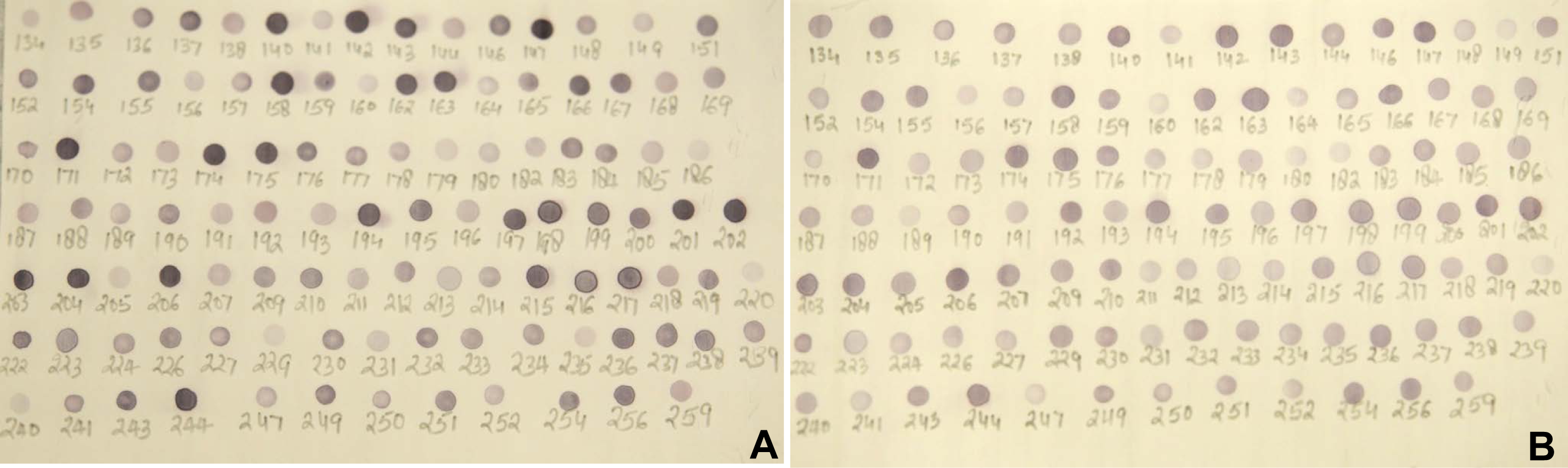

Supplement: Additional file 2 — Dot-blot hybridization of clones amplified by the adaptor-specific nested PCR. Denatured PCR products were dotted onto a positively charged nylon membrane, hybridized with enriched subtracted and unsubtracted DIG-labelled cDNA probes and analyzed for differential screening. Positive dots were identified as those that had different signal intensities on the membrane. Dots with similar intensities were considered to be non-differentially expressed transcripts (A) dots of hybridized with enriched subtracted forward cDNA probes, (B) dots hybridized with unsubtracted cDNA probe controls. [file 13567_2014_101_MOESM2_ESM.tiff]
